# SlimDeblurGAN-Based Motion Deblurring and Marker Detection for Autonomous Drone Landing

**DOI:** 10.3390/s20143918

**Published:** 2020-07-14

**Authors:** Noi Quang Truong, Young Won Lee, Muhammad Owais, Dat Tien Nguyen, Ganbayar Batchuluun, Tuyen Danh Pham, Kang Ryoung Park

**Affiliations:** Division of Electronics and Electrical Engineering, Dongguk University, 30 Pildong-ro 1-gil, Jung-gu, Seoul 04620, Korea; noitq.hust@gmail.com (N.Q.T.); lyw941021@dongguk.edu (Y.W.L.); malikowais266@gmail.com (M.O.); nguyentiendat@dongguk.edu (D.T.N.); ganabata87@gmail.com (G.B.); parkgr@dongguk.edu (K.R.P.)

**Keywords:** unmanned aerial vehicle, autonomous landing, deep-learning-based motion deblurring and marker detection, network slimming, pruning model

## Abstract

Deep learning-based marker detection for autonomous drone landing is widely studied, due to its superior detection performance. However, no study was reported to address non-uniform motion-blurred input images, and most of the previous handcrafted and deep learning-based methods failed to operate with these challenging inputs. To solve this problem, we propose a deep learning-based marker detection method for autonomous drone landing, by (1) introducing a two-phase framework of deblurring and object detection, by adopting a slimmed version of deblur generative adversarial network (DeblurGAN) model and a You only look once version 2 (YOLOv2) detector, respectively, and (2) considering the balance between the processing time and accuracy of the system. To this end, we propose a channel-pruning framework for slimming the DeblurGAN model called SlimDeblurGAN, without significant accuracy degradation. The experimental results on the two datasets showed that our proposed method exhibited higher performance and greater robustness than the previous methods, in both deburring and marker detection.

## 1. Introduction

Unmanned aerial vehicles (UAVs) or drones are successfully used in several industries. They have a wide range of applications such as surveillance, aerial photography, infrastructural inspection, and rescue operations. These applications require that the onboard system can sense the environment, parse, and react according to the parsing results. Scene parsing is a function that enables the system to understand the visual environment, such as recognizing the type of objects, place of objects, and regions of object instances in a scene. These problems are the main topics in computer vision—classification, object detection, and object segmentation. Object detection is a common topic and has attracted the most interest in recent studies. In object detection, traditional handcrafted feature-based methods showed limited performance [1,2,3,4,5,6,7,8,9,10,11,12]. A competitive approach is to apply deep-learning-based methods, which have gained popularity in recent years [13,14,15,16]. However, deploying deep learning models to a UAV onboard system raises new challenges—(1) difficulties of scene parsing in cases of low-resolution or motion-blurred input, (2) difficulties of deploying the model to an embedded system with limited memory and computation power, and (3) balancing between model accuracy and execution time. Autonomous landing is a core function of an autonomous drone, and it has become an urgent problem to be solved in autonomous drone applications. Recently, deploying deep learning models to UAV systems has become more feasible, because of both the growth in computing power and the extensive studies of deep neural networks, which have achieved significant results in scene parsing tasks, such as object detection tasks (e.g., faster region-based convolutional neural network (R-CNN) [17] and single-shot multibox detector (SSD) [18]. Therefore, the topic of autonomous drone landing has attracted much research interest, and the trend is toward autonomous landing by using deep-learning-based methods for tracking a guiding marker. Several state-of-the-art (SOTA) object detectors, based on convolutional neural networks (CNNs) have been proposed and deployed successfully for marker detection in marker tracking tasks. You only look once (YOLO) models might be the most popular deep object detectors in practical applications, because the detection accuracy and execution time are well balanced. Nevertheless, those systems have low robustness and are prone to failure when dealing with low-resolution [16] or motion-blurred images [19]. Such inputs need to be preprocessed before being fed to the detector. Thus, using a combination of a few networks as a pipeline is a promising approach to achieve this goal. In addition, drone landing causes motion of the attached camera. Even if a drone has an antivibration damper gimbal, the recorded frames are affected by motion blurring, especially in the case of high-speed landing [20]. For this reason, marker detection with motion-blurred input is a critical problem that necessarily needs to be addressed.

Therefore, we propose to learn efficient motion deblurring marker detection for autonomous drone landing, through a combination of motion deblurring and object detection, and apply a slimming deblurring model to balance the system speed with accuracy, on embedded edge devices. To this end, we trained the DeblurGAN network on our synthesized dataset and then pruned the model to obtain the slimmed version, SlimDeblurGAN. Moreover, we trained a variant of the YOLO detector on our synthesized dataset. Finally, we stacked SlimDeblurGAN and the detector, and then evaluated the system on a Desktop PC and a Jetson board TX2 environment. This research was novel compared to previous studies, in the following four ways:

- This is one of the first studies on simultaneous deep-learning-based motion deblurring and marker detection for autonomous drone landing.
- The balance of accuracy and processing speed is critical when deploying a marker tracking algorithm on an embedded system with limited memory and computation power. By proposing a dedicated framework for pruning the motion deblurring model, our proposed SlimDeblurGAN acquires the real-time speed on embedded edge devices, with high detection accuracy.
- Through an iterative performance of channel pruning and fine-tuning, our proposed SlimDeblurGAN showed a lower computing complexity but a higher accuracy of marker detection, compared to the state-of-the-art methods, including original DeblurGAN. The SlimDeblurGAN generator uses batch normalization, instead of instance normalization and imposes sparsity regularization. By performing channel pruning on the convolutional layers of the generator, SlimDeblurGAN has a more compact and effective channel configuration of the convolutional layers. Furthermore, it has a smaller number of trainable parameters than DeblurGAN. Thus, its inference time is shorter than that of the original DeblurGAN, with a small degradation of accuracy.
- The codes of pruning framework for slimming DeblurGAN, SlimDeblurGAN, and YOLOv2, two synthesized motion-blurred datasets, and trained models are available to other researchers through our website (Dongguk drone motion blur datasets and the pretrained models. http://dm.dgu.edu/link.html), for fair comparisons.

## 2. Related Works

There are numerous studies on autonomous drone landing, which can be classified into two types—those not considering motion blurring and those considering motion blurring.

Not considering motion blurring: At the initial stages, researchers considered objects on the runway with a lamp to guide the UAV, to determine a proper landing area. Gui et al. [1] proposed a vision-based navigation method for UAV landing, by setting up a system in which a near-infrared (NIR) light camera was integrated with a digital signal processing processor and a 940-nm optical filter was used to detect NIR light-emitting diode (LED) lamps on the runway. Their method had a significant advantage, i.e., it could not only work well in the daytime but also at nighttime. However, this method required a complicated setup of four LEDs on the runway. In addition, this method could only be performed in a wide area. Therefore, it failed to operate in narrow urban landing areas. Forster et al. [2] proposed a landing method including generating a 3D terrain depth map from the images captured by a downward-facing camera, and determining a secure area for landing.

It was completely proven that this method could work well in both indoor and outdoor environments. Nevertheless, the depth estimation algorithm was only tested at a maximum range of 5 m, and this method exhibited a slow processing speed. Two limitations of markerless methods are the difficulty of spotting a proper area for landing and the requirement of complicated setups for the landing area.

To solve these problems, marker-based methods were proposed. According to the type of features used, marker-based methods could be categorized into two kinds—handcrafted feature-based and deep feature-based methods. One of the handcrafted feature-based approaches that was robust to low-light conditions adopted a thermal camera-based method. These methods have high performance, even in nighttime scenarios, by using the emission of infrared light from a target on the ground. However, such methods require the drone to carry an additional thermal camera, as thermal cameras are not available in conventional drone systems. Other handcrafted marker-based approaches are based on visible-light cameras. Lin et al. [4] proposed a method to track the relative position of the landing area, using a single visible-light camera-based method. They used an international H-pattern marker to guide a drone landing in a cluttered shipboard environment. The characteristic of this method was that it could restore the marker from partial occlusion, and correctly detect the marker from complicated backgrounds. Moreover, they adopted the Kalman filter to fuse the vision measurement with the inertial measurement unit (IMU) sensor outputs, to obtain a more accurate estimate. Following that approach, Lange et al. [5] introduced a method to control the landing position of autonomous multirotor UAVs. They also proposed a new hexagonal pattern of landing pads, including concentric white rings on a black background and an algorithm to detect the contour rings from the landing pads. In addition, they used auxiliary sensors such as the SRF10 sonar sensor (Robot Electronics, Norfolk, UK), which accurately measured the current altitude above the ground, and the Avago ADNS-3080 optical flow sensor (Broadcom Inc., San Jose, CA, USA), which output the UAV’s current velocity. These methods have the same disadvantage as the previous one, mandatorily carrying additional hardware, such as the IMU sensor, sonar sensor, and optical flow sensor. Some previous studies investigated UAV landing on a moving platform [6,20]. These studies take account of the six-degrees of freedom (6-DOF) pose of the marker, by using special landing pads, like fiducial markers. They also investigated a landing scenario in which the markers were positioned on the deck of a ship or placed on a moving platform. Other than landing on a fixed area, this method not only solved the marker-tracking problem but also tackled the more challenging obstacle. However, it requires more calculations and the estimation of relative position between the UAV and the moving target. Hence, they used SOTA computer vision methods, including multisensor fusion, tracking, and motion prediction of landing target on the moving platform. Consequently, the limitation of such methods is the short working range, due to the limited working range of the hardware employed. In particular, a previous study adopted the fiducial AprilTag [21] marker as the landing pad, owing to its robustness in difficult situations, such as severe rotation, heavy occlusion, light variation, and low image resolution. Although this study successfully tracked the marker in daytime conditions, the maximum distance between the landing target and the UAV was only approximately 7 m.

Araar et al. [7] proposed a new solution for multirotor UAV landing, using a new landing pad and relative-pose-estimation algorithm. In addition, they adopted two filters (an extended Kalman filter and an extended H_∞) to fuse the estimated pose and the inertial measurement. Although their method was highly accurate, it required information on the inertial measurements. Additionally, only indoor environment experiments were conducted, and the maximum working range was limited, owing to the drawback of the employed AprilTag marker. A novel idea was adopted in another study, taking advantage of cloud computing to overcome the limitation of the onboard hardware [11]. Specifically, the heavy computation tasks of computer vision were transferred to a cloud-based system, and the onboard system of the UAV only handled the returned results. Barták et al. [8] introduced an adequate handcrafted marker-based method for drone landing. Handcrafted feature-based techniques, such as blob pattern recognition, were adopted to identify and recognize the landing target. Control algorithms were also employed to navigate the drone to the appropriate target area. In this way, this method worked well in real-world environments. Nevertheless, their experiments were conducted only during daytime, and the maximum detection range was limited to 2 m. In an attempt to address autonomous UAV landing on a marine vehicle, Venugopalan et al. [9] proposed a method that adopted handcrafted feature-based techniques, like color detection, shape detection, pattern recognition, and image recognition, to track the landing target. Additionally, a searching and landing algorithm and a state machine-based method, were proposed. Their method worked well, with a success rate of over 75%, even in some difficult environmental conditions like oscillatory motion associated with the landing target or wind disturbance. However, the testing distance between the landing target and the UAV in their experiments was close. Wubben et al. [10] proposed the method for accurate landing of unmanned aerial vehicles, based on ground pattern recognition. In their method, a UAV equipped with a low-cost camera could detect ArUco markers sized 56 × 56 cm, from an altitude of up to 30 m. When the marker was detected, the UAV changed its flight behavior in order to land on the accurate position where the marker was located. Through experiments, they confirmed an average offset of only 11 cm from the target position, which vastly enhanced the landing accuracy, compared to the conventional global positioning system (GPS)-based landing, which typically deviated from the intended target by 1 to 3 m. Some researchers studied the autonomous landing of micro aerial vehicles (MAVs), using two visible-light cameras [12]. They performed a contour-based ellipse detection algorithm to track a circular landing pad marker in the images obtained from the forward-facing camera. When the MAV was close to the target position, the downward-facing camera was used because the fixed forward-facing camera view was limited. By using two cameras to extend the field of view of the MAV, the system could search for the landing pad even when it was not directly below the MAV. However, this method was only tested in an indoor scenario, which limited the working range.

In order to overcome the performance limitations of the handcrafted feature-based methods, deep feature-based methods were introduced, which exhibited high accuracy and increased detection range. Nguyen et al. [13] proposed a marker tracking method for autonomous drone landing, based on a visible-light camera on a drone. They proposed a variant of YOLOv2 named lightDenseYOLO to predict the marker location, including its center and direction. In addition, they introduced Profile Checker V2 to improve accuracy. As a result, their method could operate with a maximum range of 50 m. Similarly, Yu et al. [14] introduced a deep-learning-based method for MAV autonomous landing systems, and they adopted a variant of the YOLO detector to detect landmarks. The system achieved high accuracy of marker detection and exhibited robustness to various conditions, such as variations in landmarks under different lighting conditions and backgrounds. Despite achieving high performance in terms of detection range and accuracy, these methods did not consider input images under conditions like low-resolution and motion-blurred images. In another study, Polvara et al. [15] proposed a method based on deep reinforcement learning to solve the autonomous landing problem. Specifically, they adopted a hierarchy of double-deep Q-networks that were used as high-level control policies to reach the landing target. Their experiments, however, were only conducted in indoor environments.

Recently, Truong et al. [16] proposed a super-resolution reconstruction (SR) marker detection method for autonomous drone landing, by using a combination of SR and marker-detection deep CNNs, to track the marker location. Their proposed method successfully handled the obstacle of low-resolution input. Moreover, they introduced a cost-effective solution for autonomous drone landing, as their system required only a low-cost, low-resolution camera sensor, instead of expensive, high-resolution cameras. Furthermore, their proposed system could operate on an embedded system and acquired a real-time speed. However, they did not consider the case of motion blurring in the captured image. A low-resolution image was acquired by a low-resolution camera, including the small number of pixels from the camera sensor, but the motion blurring was caused by the f-number of the camera lens and the camera exposure time. A small f-number and a large exposure time caused a large amount of motion blurring in the captured image. It is often the case that motion blurring frequently occurred in the captured image by drone camera, because the image was captured while the drone was moving or landing. Therefore, we propose a new method of motion deblurring and marker detection for drone landing, which is completely different from the previous work [16], which considers only SR of the low-resolution image by drone camera, without motion deblurring. In addition, we propose a new network of SlimDeblurGAN for motion deblurring (that is different from previous work [16]), which used deep CNN with a residual net skip connection and network-in-network (DCSCN) for SR. Considering the motion blur method: All previous methods exhibited promising solutions for autonomous landing. They conducted experiments based on various scenarios like indoor, outdoor, daytime, and nighttime, as well as difficult conditions like low light and low resolution of the input. However, the input images under the motion blur effect, which frequently occur due to the movement of the drone, were not considered in their studies. Therefore, we propose a deep-learning-based motion deblurring and marker detection method for drone landing. These research studies [13,14] were about marker detection by a drone camera and did not consider the motion blurring in the captured image, which was different from our research considering motion deblurring. The research in [20] dealt with the motion blurring in the captured image by UAV, but they did not measure the accuracy of marker detection and the processing speed on the actual embedded system for the drone. Different from this research, we measured the accuracies of marker detection by our method and compared them with the state-of-the-art methods. In addition, we measured the processing speed of marker detection by our method on the actual embedded system for the processing on the drone and compared them with the state-of-the-art methods. The research [19] studied the detection of motion-blurred vehicle logo. However, its target was only for logo detection, which was different from our research of marker detection by a drone camera. Although the method in [13,14,21] achieved a 99% accuracy for landmark or marker, based on field experiments, they assumed only the slow movement or landing of drone, which did not generate the motion blurring. However, in the actual case of drone movement or landing at normal speed, motion blurring occurred frequently, as mentioned in [20]. Table 1 presents a comparison of the proposed and previous methods.

## 3. Proposed Method

### 3.1. A. Proposed Two-Phase Framework of Motion Deblurring and Marker Detection for Autonomous Drone Landing

**Figure 1:**
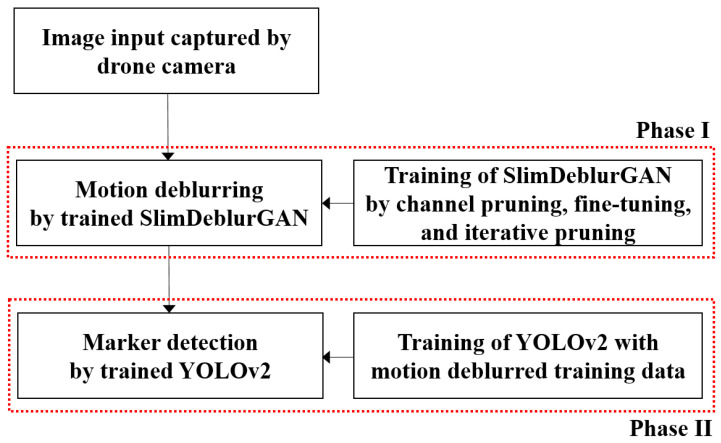
A block diagram of proposed motion deburring and marker detection system.

Our goal was to propose a model M to accurately detect a marker object in a motion-blurred image $x^{blur}$. The factor blur indicated that the image x was affected by motion blur. For that, a framework was considered, which combined two models, including a motion deblurring model that acted as a preprocessing model (P) to predict the sharp image ${\hat{y}}^{sharp}=P\left(x^{blur},\;\theta_{P}\right)$, and a follow-up marker detection model (*S*) that predicted the marker object based on the predicted sharp image $\hat{y}=S\left({\hat{y}}^{sharp},\;\theta_{S}\right)$. Here, $\theta_{P}$ is the set of trainable parameters of the preprocessing model (*P*), and $\theta_{S}$ is the set of trainable parameters of the marker detection model (*S*). This framework was promising because the motion deblurring model helped to recover the blurred input image to the sharp image, on which the detector could easily act. In addition, it had several advantages like separate independent training, guaranteed model convergence in the framework, and leveraging the SOTA models. Therefore, we proposed a two-phase framework for motion deblurring and marker detection, as shown in Figure 1. Phase I is a motion deblurring preprocessor P that uses our proposed SlimDeblurGAN model, and Phase II is the marker detector S that uses a YOLOv2 detector, which intakes the motion deblurred output from Phase I and outputs the predicted bounding box of the marker. The remainder of this section on the proposed method is organized according to the two phases.

### 3.2. Phase I: Blind Motion Deblurring by SlimDeblurGAN

Motion deblurring is a method of sharpening the blurring of an image caused by the motion of object or camera during the exposure time. Such methods are categorized into two kinds—blind and nonblind deblurring. Nonblind deblurring methods assume that the blur source is known, whereas blind deblurring methods suppose that blur source is unknown, and they estimate both a latent sharp image and blur kernels. Kupyn et al. proposed DeblurGAN [22], which is a blind motion deblurring method that achieved the SOTA performance, while being faster than its closest competitor, DeepDeblur [23], by a factor of five. In this study, we did not directly use DeblurGAN in our framework; instead, we used a slim version that was pruned from the base model DeblurGAN. The pruning process is described in Section 3.2.2. Section 3.2.1 briefly explains the original DeblurGAN.

#### 3.2.1. Blind Motion Deblurring by DeblurGAN

**Figure 2:**
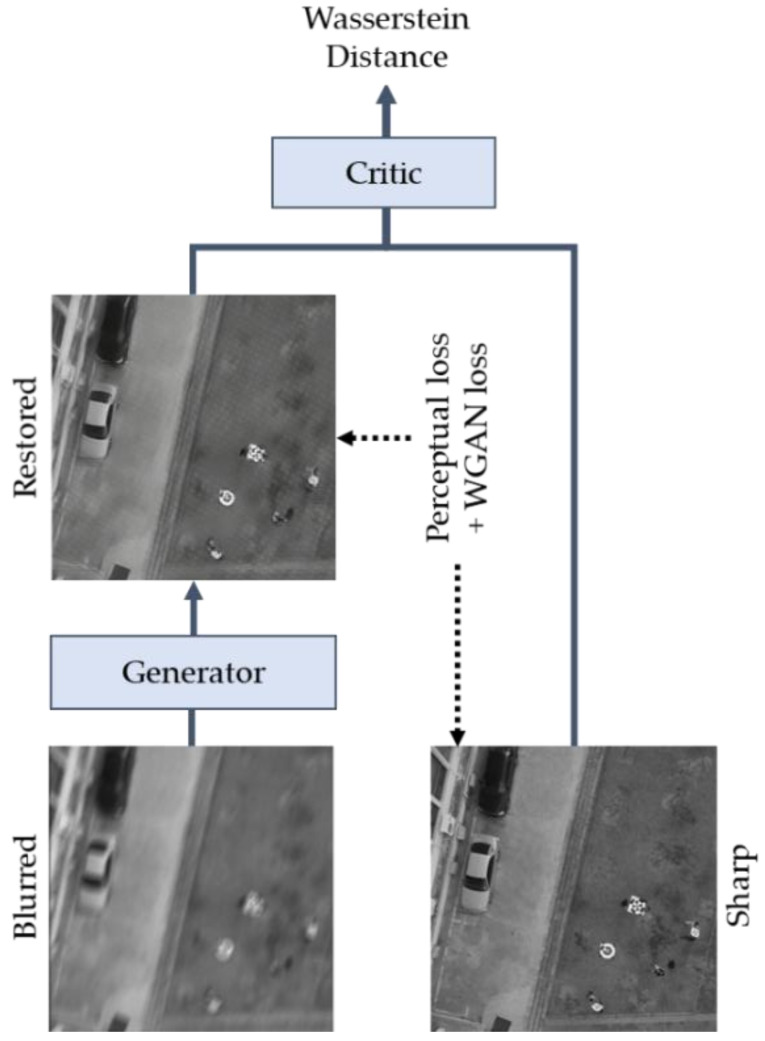
DeblurGAN architecture. The generator recovers the latent sharp image from the blurred image. The critic outputs the distance between the restored and sharp images. The total loss consists of perceptual loss and WGAN loss from the critic. After training and channel pruning, only the generator is used in Phase I.

The family of conditional generative adversarial network (cGAN) [24] was successfully applied in some image translation applications such as super-resolution [25], style transfer [26], and motion deblurring. DeblurGAN was designed as a cGAN using the Wasserstein GAN gradient penalty (WGAN-GP) [27] as the critic function. Training GAN models required the procedure of finding a Nash equilibrium of a noncooperative two-player game [28]. Sometimes the gradient descent does this and at others, it does not, and no good equilibrium-finding algorithm was reported yet. These difficulties led to a novel idea, WGAN, which used an alternative objective function—using the Wasserstein distance instead of the traditional Jensen–Shannon distance, because it helped to increase the training stability [29]. Gulrajani et al. [27] then proposed WGAN-GP, which was an updated version, robust to the choice of generator architecture. For this crucial reason, DeblurGAN adopted WGAN-GP as a critic function, which allowed DeblurGAN to use a lightweight CNN architecture as a generator. The DeblurGAN architecture included a generator network and a critic network, as shown in Figure 2.

The generator was the same as that proposed by Johnson et al. [30] for style transfer tasks. It contained two convolution blocks, nine residual blocks [31] (ResBlocks), and two transposed convolution blocks. Each ResBlock had a convolution layer, instance normalization layer [32], and rectified linear unit (ReLU) [33] activation. In contrast to the original one proposed by Johnson et al. [30], the DeblurGAN generator had an additional global skip connection, which was referred to as ResOut. The detailed information of the generator architecture is shown in Table 2. The critic network architecture was identical to that of PatchGAN [34].

DeblurGAN loss included content loss and adversarial loss, as shown in Equation (1):

(1) $$L=L_{GAN}+\lambda \;\cdot L_{X},$$

where the total loss L is the sum of the adversarial loss $L_{GAN}$ and content loss $L_{X}$; the coefficient λ denotes the balance between the two types of losses and it was set to 100 in all experiments.

Adversarial loss was described as:

(2) $$L_{GAN}=\overset{N}{\underset{n=1}{\sum }}-D_{\theta_{D}}\left(G_{\theta_{G}}\left(X^{blur}\right)\right).$$

$D_{\theta_{D}}$ and $G_{\theta_{G}}$ are the discriminator and generator, respectively. $\theta_{D}$ and $\theta_{G}$. are the trainable parameters of the discriminator and generator, respectively.

Content loss was the perceptual loss [30], which was defined as:

(3) $$L_{X}=\frac{1}{W_{i,j}H_{i,j}}\overset{W_{i,j}}{\underset{x=1}{\sum }}\overset{H_{i,j}}{\underset{y=1}{\sum }}{\left(\phi_{i,j}{\left(X^{sharp}\right)}_{x,y}-\phi_{i,j}{\left(G_{\theta_{G}}\left(X^{blur}\right)\right)}_{x,y}\right)}^{2},$$

where $\phi_{i,j}$ is the feature map obtained by the $i^{th}$ convolution within the VGG19 network, pretrained on ImageNet [35], and $W_{i,j}$ and $H_{i,j}$ are the width and height of the feature maps, respectively. $\theta_{G}$ is the set of trainable parameters of the generator ($G_{\theta_{G}})$.

The authors proved experimentally that without this perceptual loss or without replacing the perceptual loss with a simple mean square error (MSE), the network did not converge to a meaningful state [22].

#### 3.2.2. Proposed SlimDeblurGAN

As we adopted a two-phase process, the execution time of the proposed framework $t_{overall}$ was the sum of the time for each model element in two phases, including the processing time of motion deblurring $t_{P}$, and that of marker detection $t_{D}$, as illustrated in Equation (4).

(4) $$t_{overall}=t_{Phase\;I}+t_{Phase\;II}=\;t_{P}+t_{D}.$$

In Equation (4), the processing time of detection was much shorter than that of the motion deblurring model. Informatively, the processing time of the YOLOv2 detector was shorter than that of DeblurGAN, by almost 17 times. Therefore, a slimmed deblurring model P was crucial to reduce the execution time, and thus increased the execution speed of the overall system.

**Figure 3:**
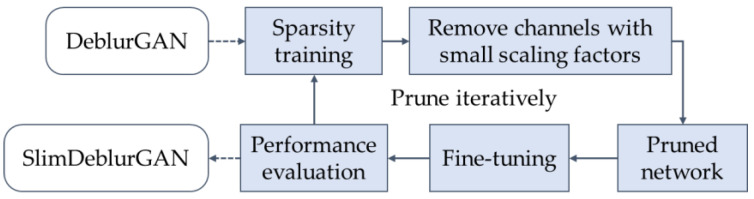
Iterative procedure of pruning of the DeblurGAN model through sparsity training and channel pruning for SlimDeblurGAN.

Considering the recently proposed methods for network lightening, such as using MobileNet [36] as a backbone, manually reducing the number of layers, network slimming [37], knowledge distillation [38], and dynamic computation [39], we settled on the network slimming proposed by Liu et al. [37]. This was a novel learning scheme for learning efficient convolutional networks, which reduced the model size, decreased the run-time memory footprint, and lowered the number of computing operations, without compromising accuracy. Essentially, the network slimming method is a technique to learn more compact CNNs. It directly imposed sparsity-induced regularization on the scaling factors in batch normalization layers, and the unimportant channels could thus be automatically identified during training, which could then be pruned. It is conceptually easy to understand; however, proposing a framework that can prune well for every network is challenging, as each network has its different components and irregular network architecture. Liu et al. applied a network slimming method to prune image classifier CNNs [37]. Zhang et al. [40] then extended the scheme to a coarse-grained method and successfully applied it to a slim YOLOv3 network. Inspired by the works of Liu and Zhang et al. [37,40], we proposed a model pruning procedure for pruning the DeblurGAN model to obtain SlimDeblurGAN, as shown in Figure 3 and Table 3.

Adapting DeblurGAN for model pruning. Our goal was to reduce the processing time of the proposed system by reducing the execution time of Phase I. This phase was a motion deblurring task that could be performed by the generator of DeblurGAN. In addition, only the generator was kept and employed in the testing time. Therefore, we conducted the process of training and pruning DeblurGAN to obtain SlimDeblurGAN. To this end, we proposed a slimming framework for pruning only the generator, while keeping the remaining part of the DeblurGAN. We adapted DeblurGAN for the pruning process, by modifying the generator architecture. In more detail, the original DeblurGAN generator used the instance normalization layer; however, we replaced all instance normalization layers with batch normalization (BN) layers and imposed L1 regularization on the BN layers.

Channel-level sparsity training of DeblurGAN. Sparsity could be implemented at different levels, such as the weight level, kernel level, layer level, or channel level. Among these levels, the channel level provided the best tradeoff between flexibility and ease of implementation. The idea of channel-level sparsity training was to adopt a scaling factor γ for each channel, where |γ| denoted the channel importance, and then to jointly train the network weights and the scaling factors. As there are some identical properties between desired architecture and the BN architecture, the implementation of channel-level sparsity could leverage the BN layer.

Specifically, the BN layer was formulated as shown in the following equations:

(5) $$\hat{z}=\frac{z_{in}-\mu_{Β}}{\sqrt{\sigma_{Β}^{2}+\epsilon }},$$

(6) $$z_{out}=\gamma \hat{z}+\beta ,$$

where $z_{in}$, $\mu_{Β}$, and $\sigma_{Β}^{2}$ are respectively the input features, mean, and variance of input features in a minibatch, and γ and β denote the trainable scale factor and bias (scale and shift), respectively. The trainable scale factor γ in the BN layer could be adopted as an indicator of channel importance. To impose sparsity regularization, a sparsity-induced penalty was added to the training objective ($loss_{network}$), which was given as

(7) $$L=loss_{network}+\lambda \underset{\gamma }{\sum }f\left(\gamma \right),$$

where f(γ)=|γ| and λ denotes the penalty factor. γ is the trainable scale factor.

Channel pruning. To achieve this goal, we adopted an expected pruning ratio r, which was an expected ratio of the number of expected pruned channels to the overall feature channels. Based on r and the sorted list of all |γ|, a global threshold $\hat{\gamma }$ was experimentally obtained, which determined whether a channel of the feature map was to be pruned. Feature channels, whose scaling factors were smaller than the threshold $\hat{\gamma }$ were pruned.

Fine-tuning SlimDeblurGAN. After channel pruning, model fine-tuning was necessary to compensate for temporary accuracy loss, which showed an even higher accuracy than the model without fine-tuning.

Iterative pruning. The repetition of the pruning procedure (as shown in Figure 3) helped to avoid over-pruning, which caused the pruned model degradation and could not be recovered by fine-tuning or performing more pruning steps.

#### 3.2.3. Summarized Differences between the Original DeblurGAN and Proposed SlimDeblurGAN

We summarized the differences between the original DeblurGAN and the proposed SlimDeblurGAN, as follows.

- SlimDeblurGAN generator uses batch normalization instead of instance normalization and imposes sparsity regularization.
- By performing channel pruning on convolutional layers of the generator, SlimDeblurGAN has a more compact and effective channel configuration of the convolutional layers and it has a smaller number of trainable parameters than DeblurGAN. Thus, its inference time is shorter than that of the original DeblurGAN, with a small degradation of accuracy, as shown in the experimental section.

### 3.3. Phase II: Marker Detection by YOLOv2 Detector

**Figure 4:**
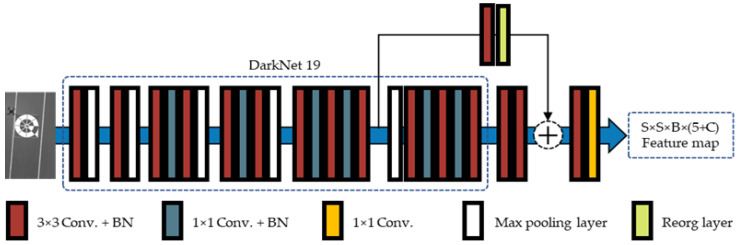
YOLOv2 backbone convolutional neural networks (CNN) architecture. The backbone network is a combination of the first 18 layers of Darknet19 and the YOLOv2 header. The header includes four convolutional layers and a Reorg layer. The Reorg layer refers to reorganization, which manipulates the output feature map of the 13th convolution of Darknet19 to obtain a reorganized feature map. The Concat layer concatenates the reorganized feature map and the output feature map of the 20th convolution. The final output is the feature map of shape S × S × B × (5 + C).

Deep object detectors have attracted much interest in recent years. Several SOTA deep object detectors were proposed, including Fast R-CNN [41], Faster R-CNN [17], R-FCN [42], RetinaNet [43], SSD [18], and the YOLO series (YOLO [44], YOLOv2 [45], and YOLOv3 [46]). According to the adoption of extra region proposal modules, these could be categorized into two classes—two-stage and one-stage detectors. In particular, the YOLO series, which were one-stage detectors, were widely adopted in practical applications [44,45,46], because the accuracy and speed were well-balanced. Therefore, we adopted YOLOv2 using Darknet19 of Figure 4 as the main part of the feature extraction, as a marker detector in Phase II.

In YOLOv2 [45], the Reorg layer was used to reshape the feature map, so that the width and height of the input feature map matched the other output feature map, and these two feature maps could be concatenated together. However, our marker datasets were quite different, compared to other object detection datasets like COCO [47], because the number of classes to be detected was only one (the marker), and its size varied from small to large, according to the height of the drone above the landing area. Therefore, it was necessary to adapt YOLOv2 to remove the redundant computations. First, we considered the anchor boxes. As the marker was a circle-based shape, the ground truth bounding boxes were theoretically square. However, the image size of our dataset was 1280 × 720 pixels, and in training and testing, the input was resized to 320 × 320 pixels by bilinear interpolation [48]. Hence, the height-to-width ratio of the marker was changed to a certain ratio. Therefore, instead of choosing the anchor boxes by hand or using anchor boxes obtained from other datasets, we normalized all ground truth bounding boxes of the training dataset and clustered them through K-means clustering with a distance metric, to obtain the proper anchor boxes for our dataset. The number of anchor boxes and their sizes could be determined by the elbow curve method on the graph of the number clustering and the IoU threshold. Second, we set the input size for the backbone network of 320 × 320 pixels as close to the output of Phase I, 256 × 256 pixels.

According to the YOLOv2 network [45], the output image was represented as S × S grids, and S was defined as 10. Therefore, the output feature map of the S × S grids was 10 × 10. B was the number of anchor boxes (in our experiment, B was defined as 3). In detail, each grid could have three anchor boxes for representing the detected object [45]. The output shape of the feature map of the YOLOv2 network was S × S × B × (5 + C) [45]. Here, “5” meant center x, center y, width, height, and confidence of one anchor box. In addition, C was the number of class probability (in our case, C was one because there was only one marker class) of one anchor box. Consequently, the output shape of the feature map of the YOLOv2 network (S × S × B × (5 + C)) became 10 × 10 × 3 × 6 in our case.

## 4. Experimental Results

### 4.1. Experimental Environment and Datasets

As there was no open dataset of blurred images acquired from landing drones, we synthesized images from the Dongguk drone camera database version 2 (DDroneC-DB2) [13] as the synthesized motion blur drone database 1 (SMBD-DB1), and we also obtained the real motion blur drone database 1 (RMBD-DB1), which contained real motion blur in the wild.

Training and testing were performed based on a two-fold cross-validation method on these two databases. All subsets were equally distributed to training and testing per fold. In the 1st fold validation, half of the images in the SMBD-DB1 were used for training, and the other half was for testing. This procedure was repeated by exchanging the training and testing data with each other in the 2nd fold validation. The average accuracy from the two-fold validations was determined as the final accuracy. The same rule was also applied to RMBD-DB1. The details of these two datasets are presented in the following section.

**Figure 5:**
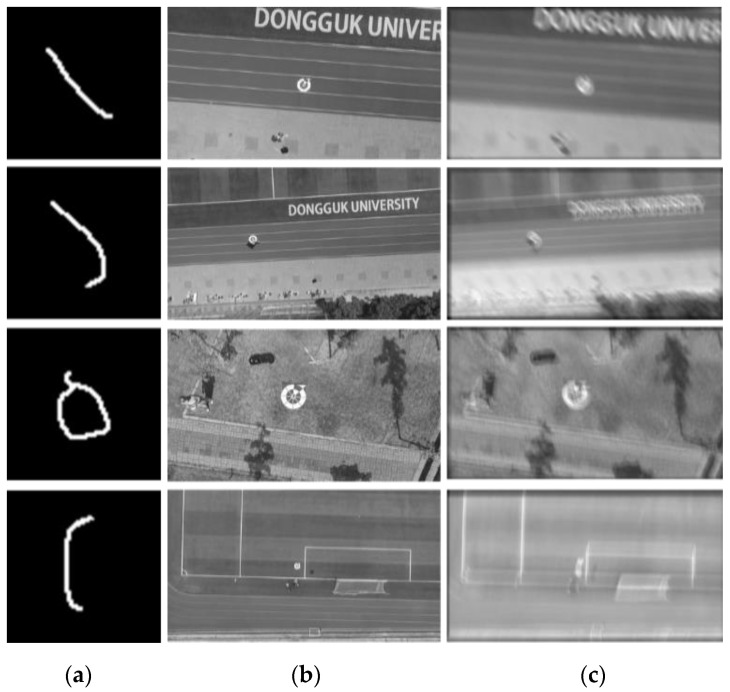
Examples of SMBD-DB1. (**a**) Synthetically generated kernels, (**b**) ground truth (original images), and (**c**) motion-blurred images.

SMBD-DB1: This dataset was generated by following the idea proposed by Kupyn et al. [22], which was based on random trajectory generation. More specifically, motion-blurred images were generated by applying the motion-blurring kernels to the original images. The motion-blurring kernels were created by applying subpixel interpolation to the trajectory vector. Each trajectory vector, which was a complex-valued vector, corresponded to the discrete positions of an object undergoing 2D random motion in a continuous domain. Figure 5 illustrates four samples of SMBD-DB1, including the motion-blurring kernels, original images, and obtained blurred images. SMBD-DB1 contains 10,642 images generated from three sub-datasets (acquired in the morning, afternoon, and evening) of DDroneC-DB2, as shown in Table 4.

**Figure 6:**
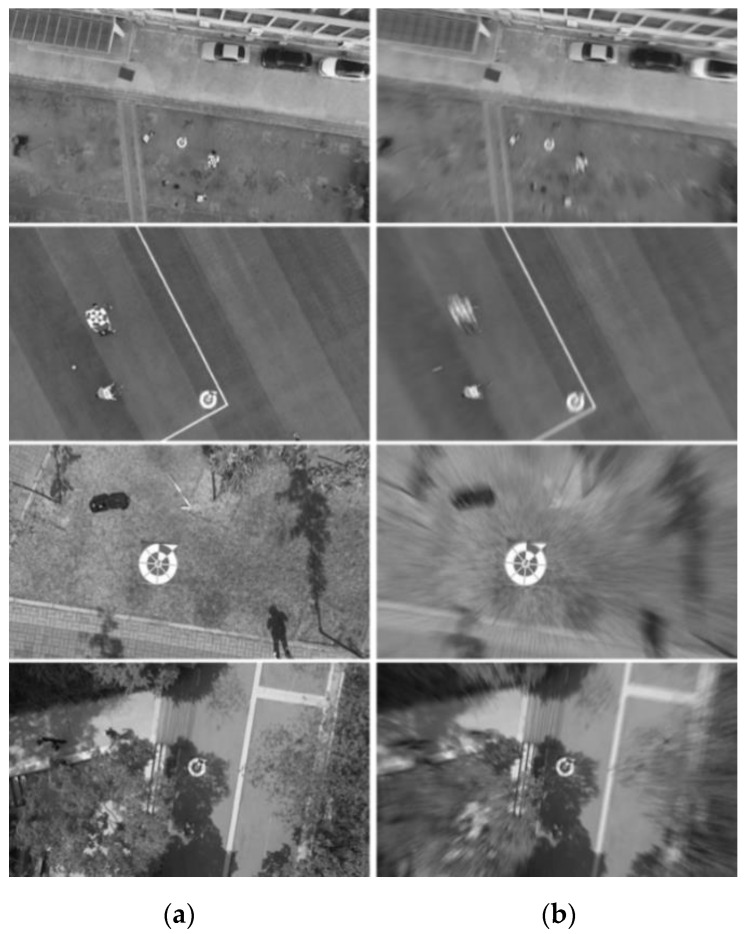
Examples of RMBD-DB1. (**a**) Ground truth (original images), and (**b**) motion-blurred images.

RMBD-DB1: For validating our method in a real-world environment, we used an additional dataset that contained real motion blur in the wild. We obtained six drone-landing videos and obtained 2991 images. The details of RMBD-DB1 are shown in Table 5. Therefore, the RMBD-DB1 included real motion of captured images by a real drone camera, and our method was expected to be performed on a real drone. The third case of Figure 6 shows the real motion blurring when the drone was fast landing.

### 4.2. Training the Proposed Method

The training of our method included two parts to train SlimDeblurGAN and YOLOv2, as explained in the following sections. All experiments, including both training and testing, were performed on a desktop computer with an Intel® Core™ i7-3770K CPU 3.5 GHz (4 cores) (Intel Corp., Santa Clara, CA, USA), 8 GB of main memory, and an NVIDIA GeForce 1070 graphics processing unit (GPU) card (1920 compute unified device architecture (CUDA) cores, and 8 GB of graphics memory) (NVIDIA Corp., Santa Clara, CA, USA).

#### 4.2.1. Training SlimDeblurGAN

We conducted a model pruning process, as mentioned in the previous section, to obtain SlimDeblurGAN. First, we created the base model and performed sparse training. Second, we repeated the iterative process of pruning and fine-tuning, until the resulting model showed a critical degradation of accuracy. We chose a model from among all pruned models throughout the pruning process, which had the best balance between accuracy and model size. Aiming to generate the base model, we replaced all instance normalizations by batch normalizations in the generator network for model pruning adaptation. As a result, we could increase the batch size to accelerate the training process. The training batch size of the DeblurGAN base model was chosen as 8, as it was the maximum batch size for which the model could be loaded in our training environment. We retained this batch size in the fine-tuning of the iterative pruning process. The number of parameters and accuracies of the resulting models obtained from the iterative channel pruning process are presented in Table 6. In this table, the peak signal-to-noise ratio (PSNR) [48] was widely used for mathematical measurements of image quality, based on the mean square error (MSE) between the pixels of ground truth image (Im(*i*,*j*)) and motion-deblurred image (Res(*i*,*j*)), as shown in Equations (8) and (9). The structural similarity measure (SSIM) index [49] could also predict the perceived quality of images. In detail, SSIM was the index showing the similarity between two images based on means, standard deviations, and the covariance of the two images.

(8) MSE =(∑j=1M∑i=1N(Im(i,j)− Res(i,j))2)2MN,

(9) $$\mathrm{PSNR}\;=10log_{10}\left(\frac{255^{2}}{MSE}\right),$$

where *M* and *N* represent the width and height of image, respectively.

**Figure 7:**
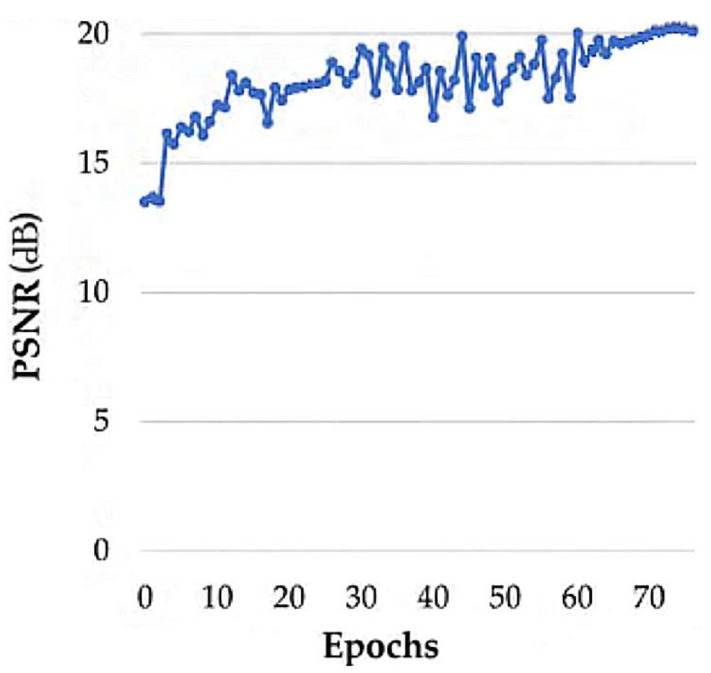
Training PSNR of SlimDeblurGAN.

The base model had 11.39 million parameters in the generator, and we performed sparse training from scratch with this model, which showed successful convergence with a PSNR of 20.59 and an SSIM of 0.49 on SMBD-DB1. We further performed channel pruning and fine-tuning with this trained base model. As a result, we obtained the first pruned model with the number of parameters in the generator reduced to 2.47 million. The fine-tuning yielded a PSNR of 21.46 and an SSIM of 0.39. Likewise, we performed the next channel pruning iterations, in which the base model was replaced with the resulting model from the previous iteration. The number of parameters in the generator after the second and third iterations decreased to 1.64 million and 1.14 million, respectively. Meanwhile, the accuracies of the resulting models degraded to 20.92 (PSNR) and 0.34 (SSIM) after the second iteration, followed by 18.16 (PSNR) and 0.26 (SSIM) after the third iteration. As shown in Table 6, the number of remaining parameters of the generator was dramatically reduced by 4.6 times after the first iteration, 6.9 times after the second iteration, and almost 10 times after the third iteration, compared to that of the base model. Although the accuracy increased after the first iteration, it decreased slightly after the second iteration, and critically degraded after the third iteration. We stopped the pruning process after the third iteration, as the observed degradation indicated over-pruning. We considered that the second pruned model had a good balance between the number of parameters and accuracy. Hence, we applied this slimmed model to the motion-deblurring phase of our system and referred to it as SlimDeblurGAN. The successful PSNR training with fine-tuning of the SlimDeblurGAN for 70 epochs appears in Figure 7.

#### 4.2.2. Training Marker Detection CNN

In an attempt to facilitate the training process of the marker detection CNN, we considered the distribution of object bounding boxes in the training dataset, in order to generate a set of anchor boxes used by YOLOv2.

**Figure 8:**
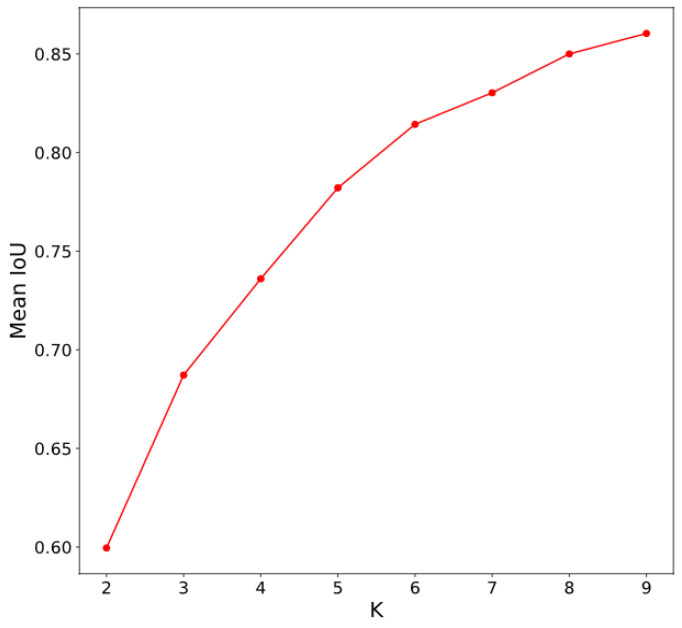
Mean IoU with respect to K for the optimal K determination.

Generating anchor boxes: We performed K-means clustering on the bounding boxes of SMBD-DB1, based on the mean IoU distance with K from 2 to 9. As shown in Figure 8, we could determine the number of clusters by the elbow curve method. As a result, 3 was the best candidate for the number of clusters. In detail, the case of K from 2 to 3 showed the largest increment in the mean IoU and we chose 3 for the number of clusters in YOLOv2.

**Figure 9:**
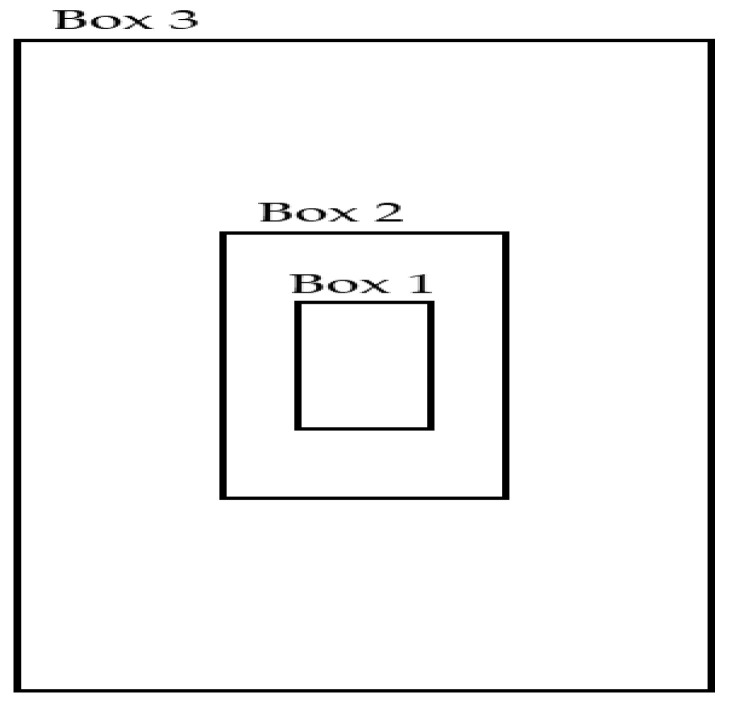
Anchor boxes.

The details of the three selected anchor boxes are shown in Table 7, and these anchor boxes are visualized in Figure 9. Notably, the actual size of the anchor boxes used in YOLOv2 depended on the grid size of the output feature map of the YOLOv2 backbone. In our experiments, we designed the backbone network to generate an output feature map grid of size 10 × 10. Therefore, the size of the anchor boxes was 10 times larger than the normalized size. The normalized bounding boxes are detailed in Table 7.

### 4.3. Testing the Proposed Method

#### 4.3.1. Accuracy of Motion Deblurring of the Proposed SlimDeblurGAN

**Figure 10:**
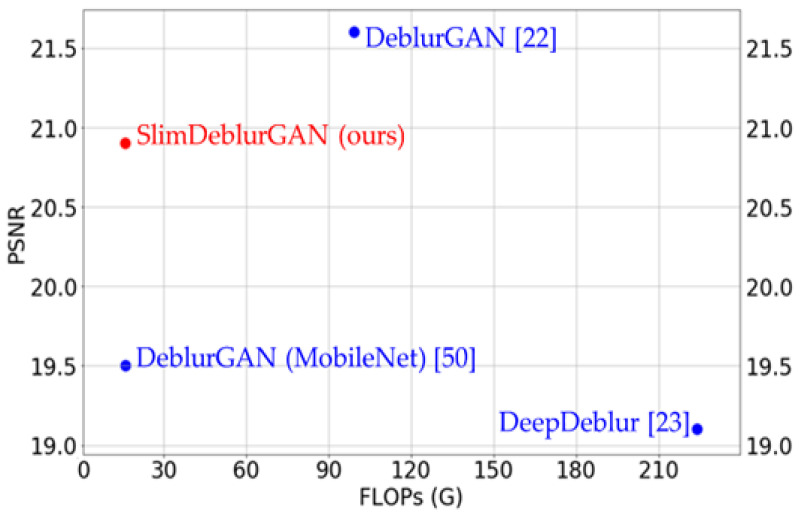
Comparison of DeepDeblur, DeblurGAN, DeblurGAN (MobileNet), and our proposed model of SlimDeblurGAN, in terms of motion deblurring accuracy and the number of FLOPs.

We conducted training and channel pruning processes to obtain SlimDeblurGAN on one fold and tested on the other fold of SMBD-DB1. In addition to measuring the testing accuracy, we measured the number of floating-point operations (FLOPs) to show the effectiveness of our proposed SlimDeblurGAN in terms of computation, compared to DeepDeblur [23], DeblurGAN [22], and DeblurGAN, using MobileNet as the backbone nets [50]. All parameters for others [22,23,50] were optimally selected by us with training data. The average results of the measurements from the two folds are presented in Table 8. The comparison of SlimDeblurGAN and the SOTA methods is also illustrated in Figure 10. As shown in this figure and table, DeblurGAN [22] showed the highest PSNR of 21.6; however, it also had a very high number of operations, at 99.3 Giga FLOPs. DeepDeblur [23] showed the lowest PSNR and highest FLOPs. Both the SlimDeblurGAN and the DeblurGAN model using MobileNet as a backbone had a small number of FLOPS, nearly one-sixth as much as that of DeblurGAN. However, DeblurGAN using MobileNet failed to maintain accuracy with a PSNR of 19.5, whereas SlimDeblurGAN had a slightly decreased accuracy, with a PSNR of 20.9. Therefore, we confirmed that our channel pruning process successfully generated a compact version of DeblurGAN with fewer FLOPs, yet high accuracy.

**Figure 11:**
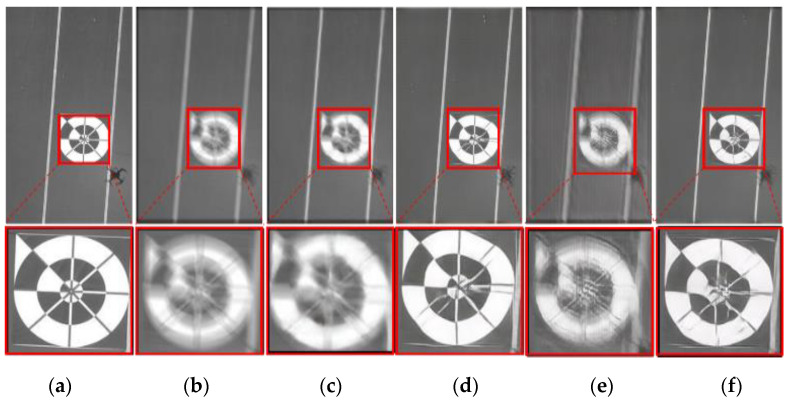
Examples of motion deblurring on SMBD-DB1. (**a**) Ground truth original image, (**b**) motion-blurred image, motion deblurring by (**c**) DeepDeblur, (**d**) DeblurGAN, (**e**) DeblurGAN (MobileNet), and (**f**) SlimDeblurGAN (ours).

Figure 11 presents examples of motion deblurring by four methods, i.e., DeepDeblur [23], DeblurGAN [22], DeblurGAN (MobileNet) [50], and SlimDeblurGAN. As shown in the figure, the results by DeblurGAN (MobileNet) and by DeepDeblur [23] were worse than those of the other methods, because the marker was still blurred and had the ghost effect as in the motion-deblurred image. However, the results obtained by DeblurGAN and SlimDeblurGAN showed sharp, non-ghost effects and recognizable markers, even by the human eye. Although the accuracy of DeblurGAN was slightly higher than that of SlimDeblurGAN, as presented in Table 8, the results obtained from both methods were almost the same in terms of perceptual comparisons.

#### 4.3.2. Accuracy of Marker Detection

**Figure 12:**
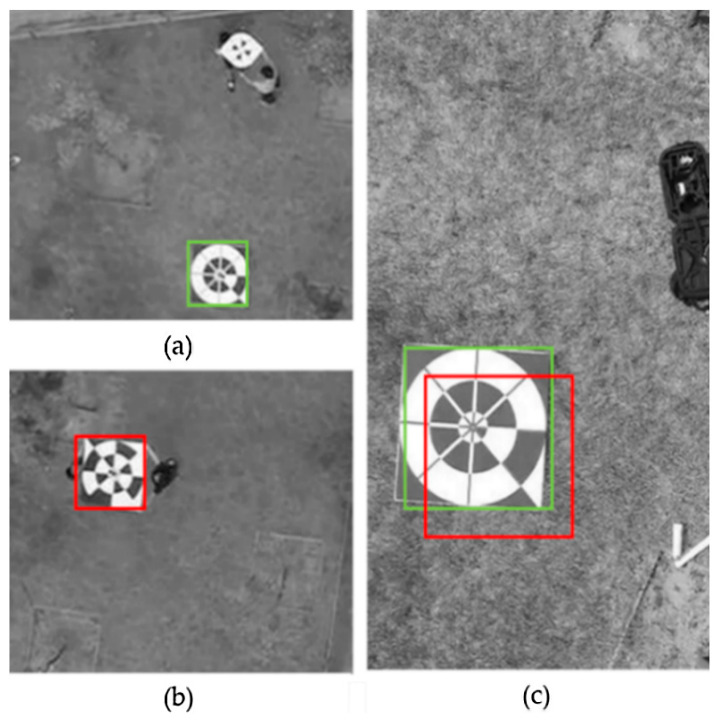
Examples of the detected results. The green boxes are ground truth labels; the red boxes are detected results. (**a**) The model cannot detect the maker on the image, and thus this case is considered as FN. (**b**) The detected result is not the marker but rather a marker-like object, and therefore this case is considered as FP. (**c**) The model can correctly detect the marker on the image; this case is considered as TP if the IoU is greater than or equal to the predefined threshold; otherwise, it is considered as FN and FP.

As the most common method of evaluating the performance of object detection system is to analyze the precision, recall, and F1 score at different IoU thresholds, we also evaluated our system in this way. These metrics were based on true positive (TP), false positive (FP), true negative (TN), and false negative (FN). In our study, TP, FP, TN, and FN could be determined by the following case studies of detection results:

Case 1: The system could not detect the marker on the image. We considered this case to be FN, as presented in Figure 12a.

Case 2: The detected object was not the marker but rather a marker-like object (i.e., wrong detection). We considered this case to be FP, as shown in Figure 12b.

Case 3: The marker was detected by the system, as illustrated in Figure 12c; we considered the IoU between the detected bounding box and the ground truth bounding box. We compared the IoU score with the predefined threshold. If the IoU was greater than or equal to the predefined threshold, this case could be considered as TP; otherwise, it could be considered to be FP and FN.

Following these definitions, we counted the number of true positives (Num. of TP), the number of false positives (Num. of FP), and the number of false negatives (Num. of FN) on the testing dataset. As a result, the accuracies could be calculated based on Equations (10)–(12).

(10) $$\mathrm{Precision}\;=\frac{\mathrm{Num}.\;\mathrm{of}\;\mathrm{TP}}{\mathrm{Num}.\;\mathrm{of}\;\mathrm{TP}\;+\;\mathrm{Num}.\;\mathrm{of}\;\mathrm{FP}},$$

(11) $$\mathrm{Recall}\;=\frac{\mathrm{Num}.\;\mathrm{of}\;\mathrm{TP}}{\mathrm{Num}.\;\mathrm{of}\;\mathrm{TP}\;+\;\mathrm{Num}.\;\mathrm{of}\;\mathrm{FN}},$$

(12) $$F1\;\mathrm{score}\;=2\cdot \frac{\mathrm{Precision}\;\cdot \mathrm{Recall}}{\mathrm{Precision}\;+\;\mathrm{Recall}}.$$

Precision reflected the proportion of correct detections out of the total number of detections, whereas recall indicated the proportion of correct detections to the total number of ground truth marker boxes in the testing dataset. There was a trade-off between precision and recall. If the model learned to predict with high precision, it tended to overfit, which caused a reduction in the recall. In contrast, if the model learned to be able to predict all markers in the dataset for a high recall, it could be more general in marker detection, which caused underfitting and degraded precision. The higher the precision, the lower recall, and vice versa. The F1 score turned out to be a better metric, which was based on precision and recall, as shown in Equation (12). In our experiments, we evaluated the detection performance on five methods—(1) YOLOv2, (2) a combination of DeepDeblur and YOLOv2, (3) a combination of DeblurGAN and YOLOv2, (4) a combination of a modified version of DeblurGAN that used MobileNet as the backbone, and YOLOv2, and (5) our proposed method—a combination of SlimDeblurGAN and YOLOv2. The measurements were conducted at different IoU thresholds on both synthesized motion-blur datasets. All parameters for other methods [21,22,23,45,50] were optimally selected by us with training data.

**Figure 13:**
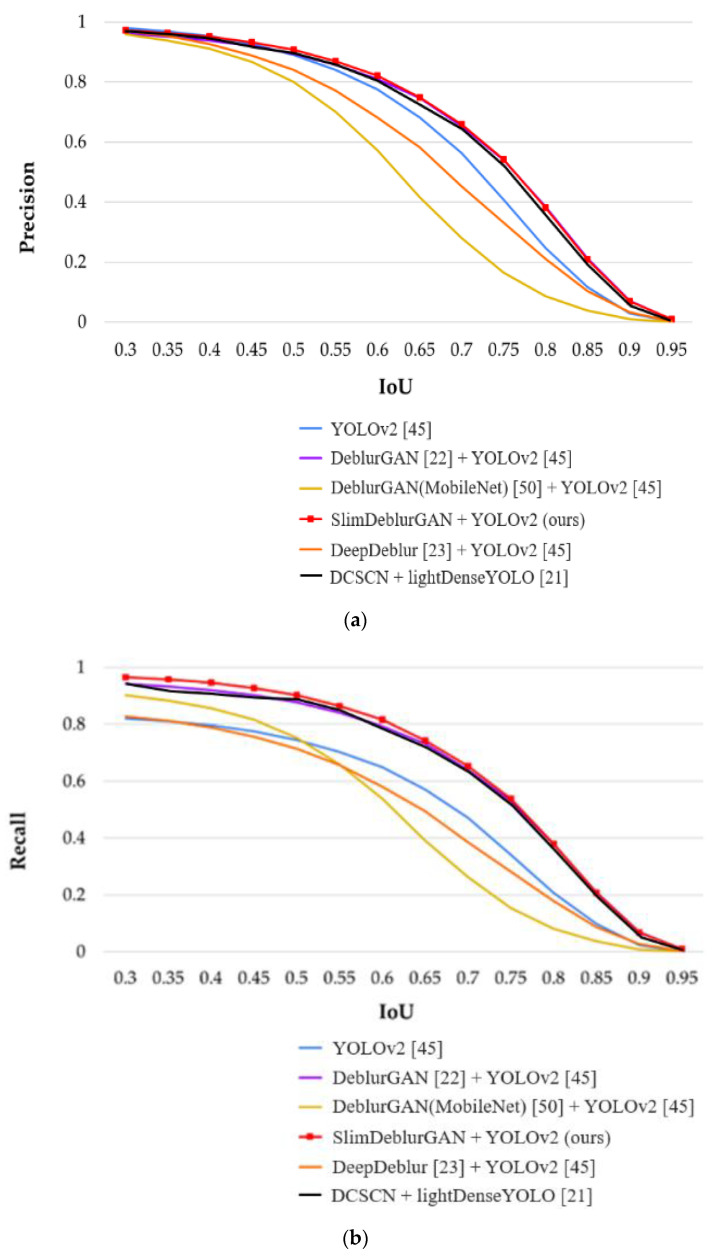

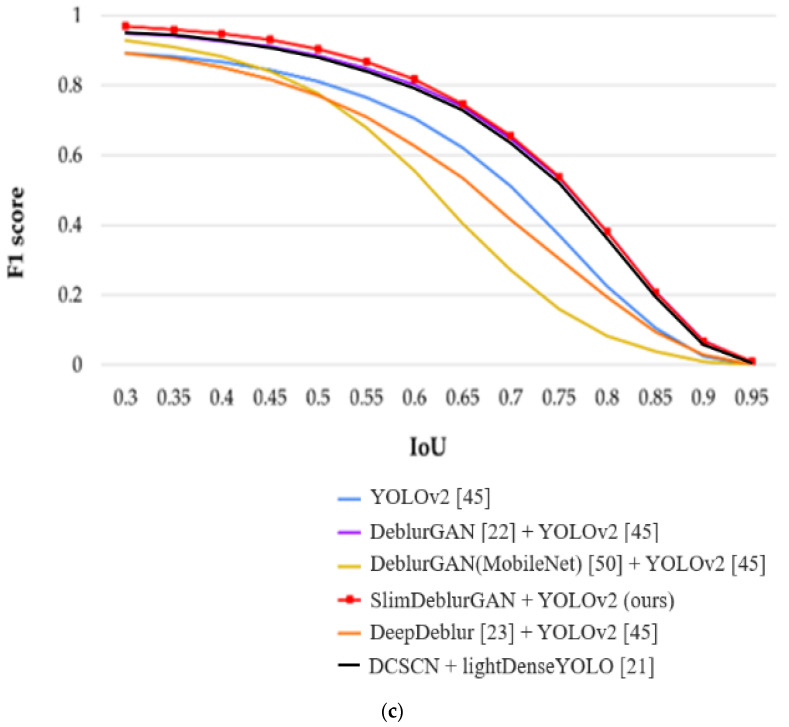
Marker detection results on SMBD-DB1 for the five methods at different IoU thresholds: (**a**) precision, (**b**) recall, and (**c**) F1 score.

Testing accuracies on SMBD-DB1: The marker detection results on SMBD-DB1 are shown in Table 9, Table 10, Table 11 and Figure 13. The precision, recall, and F1 scores of the five methods were very high at the low IoU thresholds and decreased with increasing IoU. At first glance, we could see that without any motion deblurring preprocessing, YOLOv2 exhibited low accuracies of marker detection on the motion-blurred input, as shown by the blue curves in Figure 13. The reason for this was that the motion blur strongly distorted the feature, pattern, and shape of markers in the images, making them difficult to detect. Obviously, the ghost effects in motion-blurred images were obstacles to accurately detecting the markers. Therefore, YOLOv2 could only detect the marker in images that were less affected by motion blur; otherwise, it failed. In an attempt to overcome this problem, YOLOv2 was trained directly on motion-blurred images, which could help YOLOv2 to increase its recall by learning to generalize its detection. This approach, however, decreased the precision, owing to the aforementioned tradeoff between them. Hence, the system was less accurate in distinguishing markers between marker-like objects, which caused a reduction in precision. However, with the help of highly accurate motion deblurring models such as DeblurGAN or SlimDeblurGAN, YOLOv2 obtained high precision, recall, and F1 score. Apart from DeblurGAN and SlimDeblurGAN, using DeblurGAN (MobileNet) made YOLOv2 yield lower detection accuracy than the other methods, including YOLOv2, without any deblurring preprocessing method. The failure of the DeblurGAN (MobileNet) indicated that adopting the MobileNet architecture in the backbone of the SOTA model to reduce the computation cost was not always effective. Our proposed SlimDeblurGAN showed a higher detection accuracy than the other methods, confirming that we successfully generated a compact and highly accurate motion-deblurring model of SlimDeblurGAN. In detail, the detection result by SlimDeblurGAN + YOLOv2 was slightly higher than that by the DeblurGAN + YOLOv2, as shown in Table 9, Table 10, Table 11 and Figure 13. However, the number of parameters and FLOPs of SlimDeblurGAN were less by factors of 10 and 6, respectively, than those of DeblurGAN, as shown in Table 6 and Table 8.

**Figure 14:**
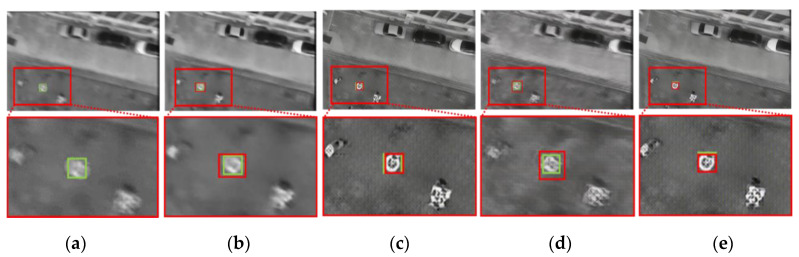
Examples of detection results on SMBD-DB1. The green boxes represent the ground truth bounding boxes and the red boxes represent the boxes detected by (**a**) YOLOv2, (**b**) DeepDeblur and YOLOv2, (**c**) a combination of DeblurGAN and YOLOv2, (**d**) a combination of DeblurGAN (MobileNet) and YOLOv2, and (**e**) a combination of SlimDeblurGAN and YOLOv2 (ours).

Figure 14 presents examples of the detection result on SMBD-DB1; the green boxes show the ground truths, and the red boxes show the detected boxes. As shown in this figure, the marker in the motion-blurred image could not be detected by YOLOv2 (without importing the additional motion deblurring stage) and could not be recognized even by the human eye. The images in Figure 14b–e were detection results on the resulting images of different deblurring methods—DeepDeblur, DeblurGAN, DeblurGAN (MobileNet), and SlimDeblurGAN, respectively.

Although the detection results of Figure 14b–e showed that the marker in the image could be successfully detected, the detected bounding boxes by different methods were different. Specifically, the detected boxes by DeblurGAN (c) and by SlimDeblurGAN (e) were closer to the ground truth than those by DeepDeblur (b) and by DeblurGAN (MobileNet) (d). From these results, we could confirm that the motion deblurring method can overcome the challenge of motion-blurred input to an object-detection system. The more accurate the motion-deblurring method, the more accurate the detection result becomes.

**Figure 15:**
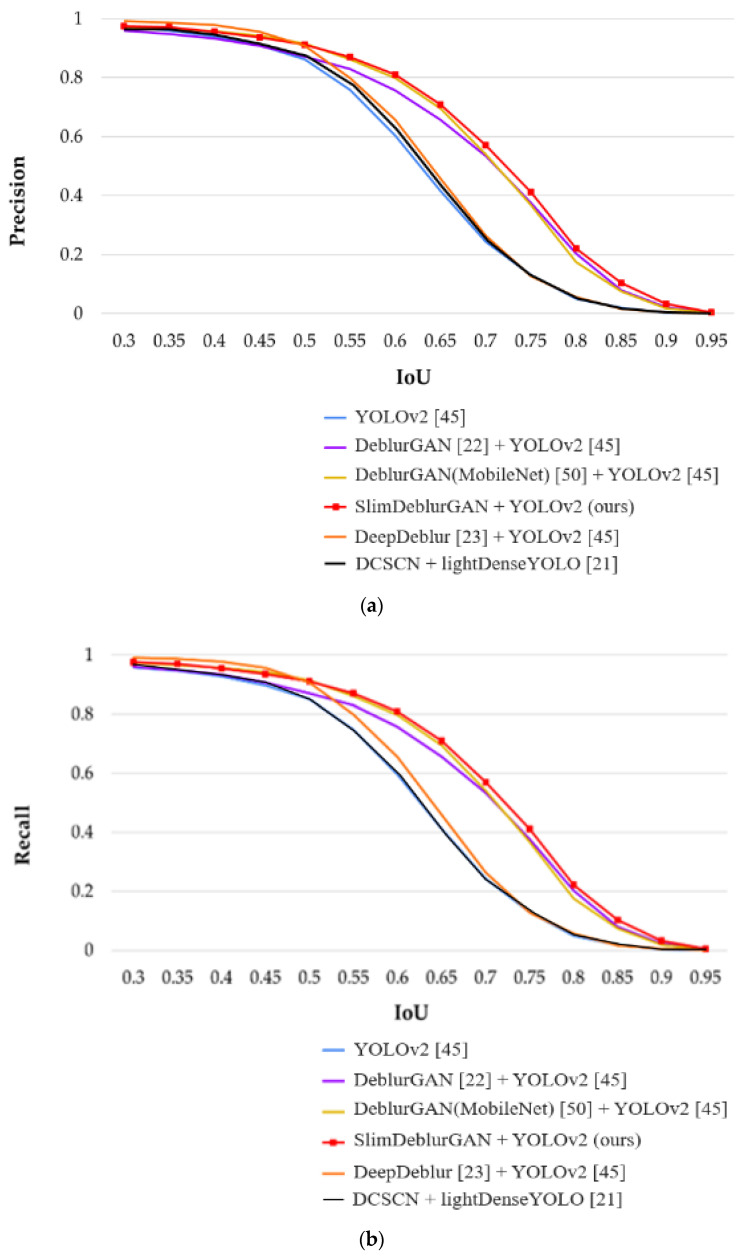

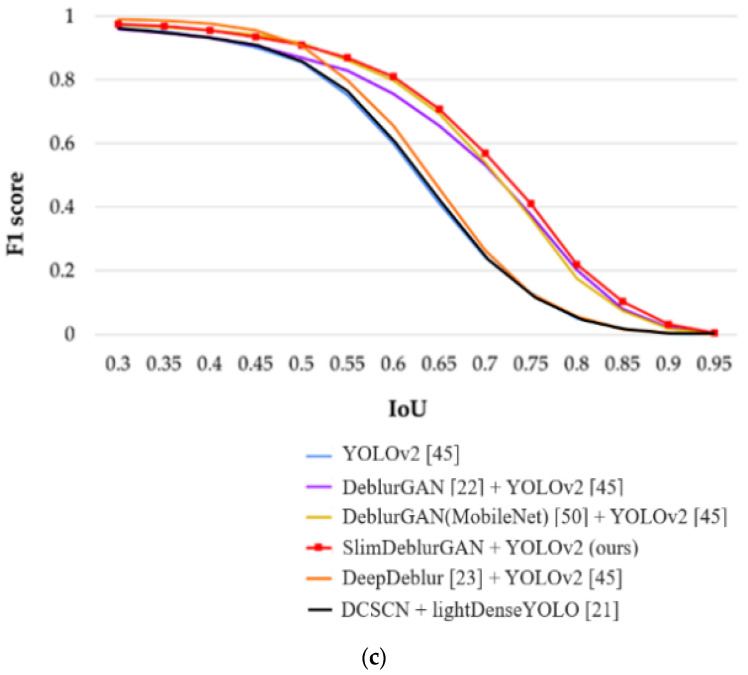
Marker detection results on RMBD-DB1 for five methods at different IoU thresholds: (**a**) precision, (**b**) recall, and (**c**) F1 score.

Testing accuracies on RMBD-DB1: Table 12, Table 13, Table 14 present the detection accuracies of precision, recall, and F1 score, respectively, on the RMBD-DB1 dataset. In addition, the comparative graphs of the experimental results are presented in Figure 15. As shown in these tables and figure, the methods combining motion deblurring and marker detection showed better detection results than the method without motion deblurring, and our proposed method yielded a better result than the SOTA methods. By testing on the RMBD-DB1 realistic motion blur dataset, we could confirm that our proposed system can work well in the real-world environment.

**Figure 16:**
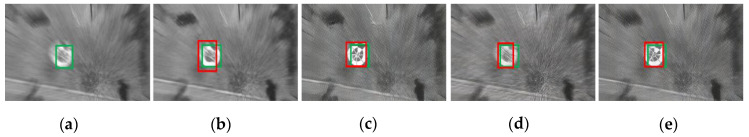
Examples of detection results on RMBD-DB1. The green boxes represent the ground truth bounding boxes and the red boxes represent the boxes detected by (**a**) YOLOv2, (**b**) DeepDeblur and YOLOv2, (**c**) a combination of DeblurGAN and YOLOv2, (**d**) a combination of DeblurGAN (MobileNet) and YOLOv2, and (**e**) a combination of SlimDeblurGAN and YOLOv2 (ours).

Figure 16 presents examples of detection results on RMBD-DB1 performed by five methods, i.e., YOLOv2, a combination of DeblurGAN and YOLOv2, a combination of DeblurGAN (MobileNet) and YOLOv2, a combination of DeepDeblur and YOLOv2, and our proposed framework combining SlimDeblurGAN and YOLOv2. The motion blur was due to the downward movement of the drone, which caused the failure of object detection by YOLOv2 (without importing the additional motion deblurring stage), as shown in Figure 16a. However, the combinations of motion deblurring models and the YOLOv2 detector successfully detected the marker, as shown in Figure 16b–d, and our method showed more accurate results of marker detection than the other methods, as shown in Figure 16e.

#### 4.3.3. Comparisons on Processing Speed and Discussion

**Figure 17:**
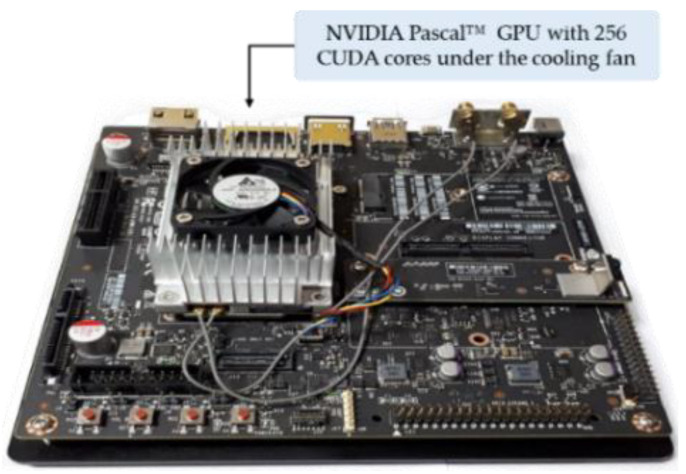
Jetson TX2 embedded system.

We measured the processing speed of our method on both an embedded system and a desktop computer. The specifications of the desktop computer are explained in Section 4.2, and a Jetson TX2 system was used as the embedded system, as shown in Figure 17. A Jetson TX2 embedded system is a fast, power-efficient device optimized for artificial intelligence (AI). It includes an NVIDIA Pascal™-family GPU (256 CUDA cores) with 8 GB of memory and features various standard hardware interfaces that facilitate integration into a wide range of products like UAVs and autonomous vehicle [51]. As the board was pre-flashed with a Linux development environment, we installed the Ubuntu 16.04 operating system, which was a convenient environment for training and testing deep learning models, as recommended by NVIDIA. Our proposed SlimDeblurGAN and YOLOv2, including the comparative algorithms were implemented in desktop computer by TensorFlow 1.14 [52], CUDA^®^ toolkit (ver. 10.0) [53], and NVIDIA CUDA^®^ deep neural network library (CUDNN) (ver. 7.6.2) [54]. These were also implemented in the Jetson TX2 system by TensorFlow 1.12 [52], CUDA^®^ toolkit (ver. 9.0) [53], and NVIDIA CUDNN (ver. 7.3) [54]. The full specifications of the Jetson TX2 embedded system are presented in Table 15.

We measured the processing time of each phase, separately. Table 16 presents the processing time per image and the FPS of four motion deblurring methods—DeepDeblur, DeblurGAN, DeblurGAN (MobileNet), and SlimDeblurGAN. The processing speed of SlimDeblurGAN on the desktop computer was extremely fast, at about 98 FPS, and it was approximately 54.6 FPS on the Jetson TX2 system. SlimDeblurGAN had the highest processing speed in both the desktop and Jetson TX2 environments.

In addition, we measured the total processing time per image of our method, including the YOLOv2 detector, as shown in Table 17. In the desktop environment, YOLOv2 archived the fast speed at 50 FPS, and it was still fast on the Jetson TX2 board with approximately 32.3 FPS. As a result, the total processing speed of our proposed method could be 33.1 FPS on the desktop computer and 20.3 FPS on the Jetson TX2 embedded system. In addition, our method was faster than the previous method, as shown in Table 17 and Table 18. YOLOv2 [45] and our previous method [21] were already applied to autonomous drone landing, and our method outperformed these algorithms, as shown in Table 9, Table 10, Table 11, Table 12, Table 13 and Table 14, Table 16, Table 18, and Figure 13 and Figure 15, which confirmed the necessity of motion blur restoration by the proposed method, for accurate marker detection. In [21], DSCN + YOLOv2 was compared with DSCN + lightDenseYOLO, which confirmed that DSCN + lightDenseYOLO proposed in [21] outperformed DSCN + YOLOv2. Therefore, we compared our method (SlimDeblurGAN + YOLOv2) with DSCN + lightDenseYOLO proposed in [21]. In Figure 14, the green boxes represent the ground truth bounding boxes, and the red boxes represent the boxes detected. There was no red box in the lower image of Figure 14a, which meant that YOLOv2 could not detect the marker in the motion blurred image. The same result could also be observed in Figure 16a. As shown in Table 9, Table 10, Table 11, Table 12, Table 13 and Table 14, and Figure 13 and Figure 15, YOLOv2 without the motion deblurring method showed lower accuracies of marker detection than our proposed method.

Although the improvement in precision by the proposed method with SMBD-DB1 was 0.5%, compared to previous work [21] (shown in Table 9), those of recall and the F1 Score by the proposed method were, respectively, 2% and 1.3% compared to previous work [21] as shown in Table 10 and Table 11. In addition, with RMBD-DB1, the improvement of precision, recall, and F1 Score of the proposed method were respectively, 11%, 12%, and 11.7%, compared to previous work [21], as shown in Table 12, Table 13 and Table 14.

## 5. Conclusions

We introduced a deep-learning-based marker detection method for autonomous drone landing, which considered motion deblurring, by proposing a two-phase framework system. To the best of our knowledge, this study was the first to consider the performance of a combination of motion deblurring and marker detection for autonomous drone landing. In addition, we considered the balance between accuracy and execution time by adopting our proposed motion-deblurring network and the real-time object detector of YOLOv2. To this end, we proposed a SlimDeblurGAN by channel pruning, to lighten the pretrained DeblurGAN model without significant degradation of accuracy, which was significantly faster than the original version. We adopted such models to our system by training from scratch and testing on our two synthesized motion-blurred datasets acquired from landing drones. We confirmed experimentally that our system could be operated well on non-uniform motion-blurred input, and it could be applied to an embedded system with low processing power. For our future work, we plan to combine the two networks of motion deblurring and marker detection into one model, including shallower layers and fewer parameters, which could reduce the processing time. In addition, we would apply our network to other applications of pedestrian detection at a distance, for intelligent surveillance camera environments, object detection in satellite images, small object detection, and moving object detection, etc.

## Figures and Tables

**Table 1:** Summary of theoretical comparisons between the proposed method and previous studies on vision-based drone landing.

Category		Type of Feature	Type of Camera	Description	Strength	Weakness
Not considering motion deblurring	Without marker	Handcrafted features	NIR light camera [1]	Detect the position of four infrared light-emitting diode (LED) lamps on the runway by using a camera attached below the head of the UAV	The maximum detection range is 450 m Works in both day and nighttime Realtime processing	Setting up the NIR lamps on the ground is difficult in various places
			Single down-facing visible-light camera [2]	Generate a local 3D elevation map of the ground environment by processing the input images from the camera sensor Estimate a safe landing spot by a probabilistic method	Can find the landing spot in an emergency case without a marker	The testing environment was ideal The maximum height for testing was 4–5 m
	With marker	Handcrafted features	Thermal images [3]	Detect letter-based marker on thermal images	Can handle various illumination challenges in marker detection	Requires an expensive thermal camera
			Single visible-light camera [4,5,6,7,8,9,10,11]	Use line segments or contour detectors to detect the marker	Requires only a single visible-light camera.	Marker detection only works in the daytime Limited detection range
			Two visible-light cameras [12]	Vision-based method for cost-effective autonomous quadrotor landing by using a landing pad	Can detect the landing pad even in the case that only a part of landing pad is visible in the camera image	Indoor experiment and limited space
		Deep features	Single visible-light camera	Use lightDenseYOLO CNN to detect the marker, and Profile Checker v2 to detect the center and direction of marker [13]	Realtime processing on the embedded system The maximum range is 50 m	The system hardware requires deep CNN support Requires a high-resolution camera
				Use a deep learning-based method for MAV autonomous landing system by detecting landmarks based on a variance of YOLO detector [14]	High-accuracy marker detection Robust to various conditions.	
				Marker detection by using double-deep Q-networks, and it navigates the drone to reach the target simultaneously [15]	Use deep reinforcement learning to solve the autonomous landing problem	Tested only in an indoor environment
			Low-resolution single visible-light camera [16]	Robust marker tracking by using a combination of SR and object detection	Requires only a single cheap and low-resolution camera Realtime processing	The system hardware requires deep CNN support
Considering motion deblurring			Single visible-light camera (**proposed method**)	Robust marker tracking by using a two-phase framework of motion deblurring and marker detection.	Can handle the non-uniform motion-blurred input images Realtime processing	The system hardware requires deep CNN support

**Table 2:** DeblurGAN generator architecture (CL means convolutional layer).

Layer Type	The Number of Filters	Size of Kernel (Height × Width)	The Number of Strides	Size of Output (Height × Width × Channel)
Input layer (Res)	-	-	-	256 × 256 × 3
1st CL	64	7 × 7	1 × 1	256 × 256 × 64
2nd CL	128	3 × 3	2 × 2	256 × 256 × 128
3rd CL	256	3 × 3	2 × 2	256 × 256 × 256
ResBlock $\left[\begin{array}{c} 3\times 3\;conv,\;1\\ 3\times 3\;conv,\;1 \end{array}\right]\times 9$	256	3 × 3	1 × 1	256 × 256 × 256
1st transpose CL	128	3 × 3	1 × 1	256 × 256 × 128
2nd transpose CL	64	3 × 3	1 × 1	256 × 256 × 64
Last CL	3	7 × 7	1 × 1	256 × 256 × 3
Output [Res + Last CL]	-	-	-	256 × 256 × 3

**Table 3:** SlimDeblurGAN: DeblurGAN generator architecture after the model pruning process (CL—convolutional layer).

Layer Type		The Number of Filters	Size of Kernel (Height × Width)	The Number of Strides	Size of Output (Height × Width × Channel)
Input layer (Res)		-	-	-	256 × 256 × 3
1st CL		10	7 × 7	1 × 1	256 × 256 × 10
2nd CL		69	3 × 3	2 × 2	256 × 256 × 69
3rd CL		251	3 × 3	2 × 2	256 × 256 × 251
ResBlock 1	4th CL	107	3 × 3	1 × 1	256 × 256 × 107
	5th CL	251	3 × 3	1 × 1	256 × 256 × 251
ResBlock 2	6th CL	59	3 × 3	1 × 1	256 × 256 × 59
	7th CL	251	3 × 3	1 × 1	256 × 256 × 251
ResBlock 3	8th CL	24	3 × 3	1 × 1	256 × 256 × 24
	9th CL	251	3 × 3	1 × 1	256 × 256 × 251
ResBlock 4	10th CL	9	3 × 3	1 × 1	256 × 256 × 9
	11th CL	251	3 × 3	1 × 1	256 × 256 × 251
ResBlock 5	12th CL	3	3 × 3	1 × 1	256 × 256 × 3
	13th CL	251	3 × 3	1 × 1	256 × 256 × 251
ResBlock 6	14th CL	3	3 × 3	1 × 1	256 × 256 × 3
	15th CL	251	3 × 3	1 × 1	256 × 256 × 251
ResBlock 7	16th CL	3	3 × 3	1 × 1	256 × 256 × 3
	17th CL	251	3 × 3	1 × 1	256 × 256 × 251
ResBlock 8	18th CL	13	3 × 3	1 × 1	256 × 256 × 13
	19th CL	251	3 × 3	1 × 1	256 × 256 × 251
ResBlock 9	20th CL	20	3 × 3	1 × 1	256 × 256 × 20
	21st CL	251	3 × 3	1 × 1	256 × 256 × 251
1st transpose CL		128	3 × 3	1 × 1	256 × 256 × 128
2nd transpose CL		64	3 × 3	1 × 1	256 × 256 × 64
Last CL		3	7 × 7	1 × 1	256 × 256 × 3
Output [Res + Last CL]		-	-	-	256 × 256 × 3

**Table 4:** Descriptions of SMBD-DB1.

Sub-Dataset	The Number of Images
Morning	4154
Afternoon	2640
Evening	3848
Total	10,642

**Table 5:** Descriptions of RMBD-DB1.

Sub-Dataset	The Number of Images
Morning	502
Afternoon	1557
Evening	932
Total	2991

**Table 6:** DeblurGAN channel pruning iterations.

Iteration	Model	The Number of Parameters of Generator	PSNR/SSIM
0	Base model (DeblurGAN)	$11.39\times 10^{6}$	20.59/0.49
1	1st pruned model	$2.47\times 10^{6}$	21.46/0.39
2	2nd pruned model (SlimDeblurGAN)	$1.64\times 10^{6}$	20.92/0.34
3	3rd pruned model	$1.14\times 10^{6}$	18.16/0.26

**Table 7:** Size of the three selected anchor boxes (* The ranges of normalized height and width are from 0 to 1, respectively).

Anchor Box	Normalized *		Size
	Height	Width	
Box 1	0.072	0.040	Small
Box 2	0.152	0.086	Medium
Box 3	0.371	0.212	Large

**Table 8:** Comparison of the number of FLOPs and accuracies of Deep Deblur [23], DeblurGAN [22], DeblurGAN using MobileNet [50], and our proposed model, SlimDeblurGAN.

Model	FLOPs (Giga)	SSIM/PSNR
DeepDeblur [23]	224.1	0.41/19.1
DeblurGAN [22]	99.3	0.40/21.6
DeblurGAN (MobileNet) [50]	16.1	0.32/19.5
SlimDeblurGAN (ours)	16	0.34/20.9

**Table 9:** Comparisons of precision on SMBD-DB1.

Methods	IoU Threshold														Average
	0.3	0.35	0.4	0.45	0.5	0.55	0.6	0.65	0.7	0.75	0.8	0.85	0.9	0.95	
YOLOv2 [45]	0.980	0.969	0.953	0.927	0.890	0.840	0.776	0.681	0.563	0.407	0.247	0.117	0.029	0.002	0.599
DeepDeblur [23] + YOLOv2	0.971	0.954	0.927	0.889	0.840	0.772	0.682	0.581	0.453	0.331	0.211	0.103	0.032	0.002	0.553
DeblurGAN [22] + YOLOv2	0.961	0.951	0.937	0.921	0.895	0.857	0.810	0.747	0.653	0.540	0.385	0.213	0.070	0.008	0.639
DeblurGAN (MobileNet) [50] + YOLOv2	0.958	0.939	0.911	0.868	0.801	0.701	0.573	0.416	0.280	0.164	0.087	0.039	0.010	0.000	0.482
DCSCN + lightDenseYOLO [21]	0.971	0.969	0.951	0.919	0.905	0.861	0.805	0.733	0.654	0.533	0.379	0.207	0.070	0.007	0.640
SlimDeblurGAN + YOLOv2 (ours)	0.972	0.964	0.952	0.933	0.908	0.870	0.821	0.748	0.658	0.542	0.382	0.208	0.068	0.009	0.645

**Table 10:** Comparisons of recall on SMBD-DB1.

Methods	IoU Threshold														Average
	0.3	0.35	0.4	0.45	0.5	0.55	0.6	0.65	0.7	0.75	0.8	0.85	0.9	0.95	
YOLOv2 [45]	0.820	0.811	0.798	0.776	0.745	0.703	0.649	0.570	0.471	0.341	0.207	0.098	0.024	0.001	0.501
DeepDeblur [23] + YOLOv2	0.827	0.812	0.789	0.757	0.715	0.657	0.580	0.495	0.386	0.282	0.180	0.088	0.027	0.002	0.471
DeblurGAN [22] + YOLOv2	0.942	0.933	0.919	0.903	0.877	0.840	0.794	0.732	0.640	0.529	0.377	0.209	0.069	0.008	0.627
DeblurGAN (MobileNet) [50] + YOLOv2	0.902	0.884	0.857	0.817	0.753	0.660	0.539	0.392	0.264	0.155	0.082	0.037	0.009	0.000	0.454
DCSCN + lightDenseYOLO [21]	0.942	0.921	0.908	0.901	0.882	0.852	0.783	0.728	0.638	0.519	0.362	0.198	0.054	0.005	0.621
SlimDeblurGAN + YOLOv2 (ours)	0.966	0.958	0.946	0.927	0.902	0.865	0.816	0.744	0.654	0.538	0.379	0.207	0.068	0.009	0.641

**Table 11:** Comparisons of the F1 Score on SMBD-DB1.

Methods	IoU Threshold														Average
	0.3	0.35	0.4	0.45	0.5	0.55	0.6	0.65	0.7	0.75	0.8	0.85	0.9	0.95	
YOLOv2 [45]	0.893	0.883	0.868	0.844	0.811	0.766	0.707	0.621	0.513	0.371	0.225	0.107	0.026	0.001	0.545
DeepDeblur [23] + YOLOv2	0.893	0.878	0.853	0.817	0.772	0.710	0.627	0.535	0.417	0.305	0.194	0.095	0.029	0.002	0.509
DeblurGAN [22] + YOLOv2	0.951	0.942	0.928	0.912	0.886	0.849	0.802	0.739	0.646	0.535	0.381	0.211	0.069	0.008	0.633
DeblurGAN (MobileNet) [50] + YOLOv2	0.929	0.911	0.883	0.841	0.776	0.680	0.556	0.404	0.272	0.159	0.084	0.038	0.009	0.000	0.467
DCSCN + lightDenseYOLO [21]	0.956	0.944	0.929	0.910	0.893	0.856	0.794	0.730	0.645	0.526	0.370	0.202	0.061	0.006	0.630
SlimDeblurGAN + YOLOv2 (ours)	0.969	0.961	0.949	0.930	0.905	0.867	0.818	0.746	0.656	0.540	0.381	0.208	0.068	0.009	0.643

**Table 12:** Comparisons of precision on RMBD-DB1.

Methods	IoU Threshold														Average
	0.3	0.35	0.4	0.45	0.5	0.55	0.6	0.65	0.7	0.75	0.8	0.85	0.9	0.95	
YOLOv2 [45]	0.972	0.959	0.940	0.910	0.862	0.758	0.604	0.416	0.245	0.131	0.050	0.019	0.003	0.000	0.491
DeepDeblur [23] + YOLOv2	0.992	0.987	0.978	0.956	0.908	0.799	0.657	0.459	0.264	0.127	0.057	0.016	0.005	0.001	0.515
DeblurGAN [22] + YOLOv2	0.961	0.949	0.933	0.908	0.871	0.830	0.757	0.657	0.534	0.376	0.203	0.080	0.021	0.002	0.577
DeblurGAN (MobileNet) [50] + YOLOv2	0.974	0.966	0.957	0.943	0.916	0.862	0.799	0.695	0.541	0.367	0.176	0.075	0.018	0.003	0.592
DCSCN + lightDenseYOLO [21]	0.971	0.956	0.939	0.909	0.870	0.771	0.625	0.431	0.262	0.125	0.055	0.018	0.004	0.001	0.496
SlimDeblurGAN + YOLOv2 (ours)	0.976	0.970	0.956	0.936	0.912	0.870	0.811	0.710	0.571	0.411	0.221	0.103	0.032	0.005	0.606

**Table 13:** Comparisons of recall on RMBD-DB1.

Methods	IoU Threshold														Average
	0.3	0.35	0.4	0.45	0.5	0.55	0.6	0.65	0.7	0.75	0.8	0.85	0.9	0.95	
YOLOv2 [45]	0.959	0.947	0.928	0.898	0.851	0.749	0.596	0.410	0.241	0.129	0.050	0.019	0.003	0.000	0.484
DeepDeblur [23] + YOLOv2	0.991	0.986	0.978	0.956	0.907	0.799	0.657	0.459	0.264	0.127	0.057	0.016	0.005	0.001	0.514
DeblurGAN [22] + YOLOv2	0.960	0.949	0.933	0.907	0.871	0.829	0.756	0.657	0.534	0.376	0.203	0.080	0.021	0.002	0.577
DeblurGAN (MobileNet) [50] + YOLOv2	0.973	0.966	0.957	0.942	0.915	0.861	0.798	0.695	0.540	0.367	0.176	0.075	0.018	0.003	0.592
DCSCN + lightDenseYOLO [21]	0.960	0.947	0.929	0.899	0.852	0.749	0.597	0.409	0.242	0.128	0.051	0.019	0.004	0.000	0.485
SlimDeblurGAN + YOLOv2 (ours)	0.975	0.969	0.955	0.935	0.911	0.870	0.810	0.709	0.570	0.411	0.220	0.103	0.032	0.005	0.605

**Table 14:** Comparisons of F1 Score on RMBD-DB1.

Methods	IoU Threshold														Average
	0.3	0.35	0.4	0.45	0.5	0.55	0.6	0.65	0.7	0.75	0.8	0.85	0.9	0.95	
YOLOv2 [45]	0.965	0.953	0.934	0.904	0.856	0.753	0.600	0.413	0.243	0.130	0.050	0.019	0.003	0.000	0.487
DeepDeblur [23] + YOLOv2	0.991	0.986	0.978	0.956	0.908	0.799	0.657	0.459	0.264	0.127	0.057	0.016	0.005	0.001	0.515
DeblurGAN [22] + YOLOv2	0.960	0.949	0.933	0.908	0.871	0.829	0.757	0.657	0.534	0.376	0.203	0.080	0.021	0.002	0.577
DeblurGAN (MobileNet) [50] + YOLOv2	0.973	0.966	0.957	0.942	0.915	0.862	0.798	0.695	0.540	0.367	0.176	0.075	0.018	0.003	0.592
DCSCN + lightDenseYOLO [21]	0.965	0.951	0.934	0.904	0.861	0.760	0.611	0.419	0.252	0.126	0.053	0.018	0.004	0.000	0.489
SlimDeblurGAN + YOLOv2 (ours)	0.975	0.970	0.956	0.936	0.911	0.870	0.810	0.709	0.570	0.411	0.220	0.103	0.032	0.005	0.606

**Table 15:** Specifications of the Jetson TX2 embedded system.

Jetson TX2 Embedded System	
**CPU**	HMP Dual Denver (2 MB L2) + Quad ARM^®^ A57 (2 MB L2)
GPU	NVIDIA Pascal™, 256 CUDA cores
Memory	8 GB
Data Storage	32 GB
Operating System	Linux for Tegra R28.1 (L4T 28.1)
Dimensions	50 mm × 87 mm

**Table 16:** Processing time (ms) per image with FPS of motion deblurring networks on two platforms.

Model	Execution Time/FPS	
	Desktop Computer	Jetson TX2 Board
DeepDeblur [23]	52/19.2	398/2.5
DeblurGAN [22]	37/27	349/2.9
DeblurGAN (MobileNet) [50]	26/38.5	215/4.7
DCSCN [21]	101/10	188/5.3
SlimDeblurGAN (ours)	10.2/98	18.3/54.6

**Table 17:** Total processing time (ms) per image by our method on two platforms.

Platform	SlimDeblurGAN	YOLOv2	Total/FPS
Desktop computer	10.2	20	30.2/33.1
Jetson	18.3	31	49.3/20.3

**Table 18:** Total processing time (ms) per image by the previous method [21] on two platforms.

Platform	DCSCN	lightDenseYOLO	Total/FPS
Desktop computer	101	20	121/8.3
Jetson	190	35	225/4.4
